# Predictors of invasive cancer of large laterally spreading colorectal tumors: A multicenter study in Japan

**DOI:** 10.1002/jgh3.12222

**Published:** 2019-07-16

**Authors:** Kiyonori Kobayashi, Shinji Tanaka, Yoshitaka Murakami, Hideki Ishikawa, Miwa Sada, Shiro Oka, Yutaka Saito, Hiroyasu Iishi, Shin‐ei Kudo, Hiroaki Ikematsu, Masahiro Igarashi, Yusuke Saitoh, Yuji Inoue, Takashi Hisabe, Osamu Tsuruta, Yasushi Sano, Hiroo Yamano, Seiji Shimizu, Naohisa Yahagi, Keiji Matsuda, Hisashi Nakamura, Takahiro Fujii, Kenichi Sugihara, the Colorectal Endoscopic Resection Standardization Implementation Working Group of the Japanese Society for Cancer of the Colon and Rectum

**Affiliations:** ^1^ Research and Development Center for New Medical Frontiers Kitasato University School of Medicine Kanagawa Japan; ^2^ Department of Endoscopy Hiroshima University Hospital Hiroshima Japan; ^3^ Department of Medical Statistics Toho University Tokyo Japan; ^4^ Department of Molecular‐Targeting Cancer Prevention, Graduate School of Medical Science Kyoto Prefectural University of Medicine Kyoto Japan; ^5^ Department of Gastroenterology Kitasato University School of Medicine Kanagawa Japan; ^6^ Endoscopy Division National Cancer Center Hospital Tokyo Japan; ^7^ Department of Gastroenterology Itami City Hospital Osaka Japan; ^8^ Digestive Disease Center Showa University Northern Yokohama Hospital Kanagawa Japan; ^9^ Department of Gastroenterology and Endoscopy National Cancer Center Hospital East Chiba Japan; ^10^ Department of Endoscopy Cancer Institute Ariake Hospital Tokyo Japan; ^11^ Digestive Disease Center Asahikawa City Hospital Hokkaido Japan; ^12^ Institute of Gastroenterology Tokyo Women's Medical University Tokyo Japan; ^13^ Department of Gastroenterology Fukuoka University Chikushi Hospital Fukuoka Japan; ^14^ Division of Gastroenterology, Department of Medicine Kurume University School of Medicine Fukuoka Japan; ^15^ Gastrointestinal Center Sano Hospital Hyogo Japan; ^16^ Department of Gastroenterology Akita Red Cross Hospital Akita Japan; ^17^ Department of Gastroenterology JR West Osaka Railway Hospital Osaka Japan; ^18^ Department of Gastroenterology, Toranomon Hospital and Cancer Center Keio University Tokyo Japan; ^19^ Department of Surgery Teikyo University School of Medicine Tokyo Japan; ^20^ Department of Gastroenterology Akasaka Endoscopic Clinic Tokyo Japan; ^21^ Gastroenterology Takahiro Fujii Clinic Tokyo Japan; ^22^ Tokyo Medical and Dental University Tokyo Japan

**Keywords:** colorectum, histopathological characteristics, large laterally spreading tumor, risk of pathological T1 cancer

## Abstract

**Background and Aim:**

Although colorectal laterally spreading tumors (LSTs) can be classified into four subtypes, the histopathological characteristics are known to differ among these subtypes. We therefore performed a logistic regression analysis to determine whether the risk of pathological T1 cancer of large colorectal LSTs can be predicted based on factors such as endoscopic findings in a large group of patients enrolled in a multicenter study in Japan.

**Methods:**

In the main study, we assessed 1236 colorectal adenomas or early cancers that were classified as LSTs measuring 20 mm or more in diameter and treated endoscopically. Logistic regression analysis was performed to determine whether factors such as the subtype of LST could be used to predict the risk of pathological T1 cancer. A validation study of 356 large colorectal LSTs was conducted to confirm the validity of the results obtained in the main study.

**Results:**

The locations and tumor diameter of the LSTs in the main study were found to differ significantly according to the LST subclassification (*P* < 0.001). The frequency of pathological T1 cancers was the highest at 36% of LST nongranular pseudodepressed type, followed by 14% of LST nongranular flat‐elevated type, 11% of LST granular nodular mixed type, and 3% of LST granular homogenous type lesions. The risk of pathological T1 cancer was significantly associated with LST subclassification and tumor diameter. The area under the curve (AUC) was high (0.743). In the validation study, the AUC was 0.573.

**Conclusions:**

In patients with large colorectal LSTs resected endoscopically, the risk of pathological T1 cancer can be predicted on the basis of the LST subclassification and tumor diameter.

## Graphical abstract

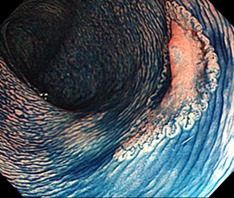

We performed a logistic regression analysis to determine whether the risk of invasive cancer of large colorectal laterally spreading tumors (LSTs) measuring 20 mm or greater and treated endoscopically can be predicted based on factors such as endoscopic findings in a large group of patients enrolled in a multicenter study in Japan. The locations and tumor diameters of the LSTs were found to differ significantly according to the LST subclassification. The risk of pathological T1 cancer was significantly associated with LST subclassification and tumor diameter.

## Introduction

Laterally spreading tumor (LST), a term describing one growth pattern of colorectal tumors, refers to colorectal tumors with a maximum diameter of 10 mm or greater and lower vertical growth than horizontal growth. The underlying disease concept was proposed by Kudo.^1^ LSTs can be classified into two types: LST granular type (LST‐G), with granules and nodules on the tumor surface, and LST nongranular type (LST‐NG), with a flat, smooth surface.^2^ LST‐G can be classified into two subgroups: LST‐G homogenous type (LST‐G‐H), with uniform granules or nodules on the tumor surface, and LST‐G nodular mixed type (LST‐G‐M), with coarse nodules on the tumor surface. LST‐NG can be classified into two subtypes: LST‐NG pseudodepressed type (LST‐NG‐PD), with a poorly demarcated, basin‐like depression in the tumor center, and LST‐NG flat‐elevated type (LST‐NG‐F), with no depression.^2^

Most colorectal LSTs are large and are associated with a low incidence of invasive carcinoma for the size. Endoscopic resection is therefore likely to be indicated for treatment. However, because LSTs are large and sessile, en bloc endoscopic mucosal resection (EMR) is difficult to perform, and endoscopic submucosal dissection (ESD) is usually carried out in Japan. Because the colon wall is thin with many curves, ESD is technically difficult to perform. Therefore, to standardize endoscopic treatment in patients with large colorectal LSTs and other tumors, the Japanese Society for Cancer of the Colon and Rectum (JSCCR) conducted a project study entitled “a multicenter collaborative study of local curability and adverse events after various endoscopic resection procedures for colorectal tumors with a maximum diameter of 20 mm or greater (prospective questionnaire survey): a cohort study of colorectal polyps measuring more than 20 mm in diameter.” The results have been reported.^3^, ^4^, ^5^, ^6^ That study focused on local curability and adverse events associated with the endoscopic resection of large colorectal tumors. Data on the endoscopic findings and histopathological characteristics of colorectal tumors measuring 20 mm or more in diameter were also collected.

The prediction of invasive cancer before treatment plays an important role in determining whether endoscopic resection is an appropriate treatment for large colorectal LSTs and is considered clinically useful. Therefore, the main objective of the present study was to clarify whether the risk of invasive cancer can be predicted on the basis of endoscopic findings in patients with large colorectal LSTs who were enrolled in a “cohort study of colorectal polyps measuring more than 20 mm in diameter.” In addition, a validation study was conducted to confirm the reliability of the results obtained in the main study.

## Methods

### *Subjects*

#### *Main study*

Eighteen middle‐ and high‐volume specialized institutions affiliated with the Colorectal Endoscopic Resection Standardization Implementation Working Group of the JSCCR participated in a cohort study of colorectal polyps measuring more than 20 mm in diameter. A total of 1845 colorectal tumors with a maximum diameter of 20 mm or greater that were resected using EMR, ESD, or snare polypectomy were registered from October 2008 to December 2010. Endoscopic resection was indicated for the treatment of intramucosal colorectal neoplasia or carcinomas with a submucosal shallow invasion of <1000 μm because the risk of lymph node metastasis is very low in such cases. Endoscopic resection is not indicated for the treatment of submucosal invasive (T1) carcinoma that is suspected to be deeper than 1000 μm, other invasive carcinomas in the colorectum, and lesions that are technically difficult to resect endoscopically and therefore require surgical resection because of factors such as the presence of circumferential tumors with a risk of stenosis after endoscopic treatment. Before endoscopic resection, the depth of invasion of large colorectal LSTs was estimated based on conventional endoscopic findings. Most lesions were also examined through chromoendoscopy, performed by spraying the mucosa with 0.2% indigo carmine dye, in combination with, if possible, pit pattern diagnosis on magnifying chromoendoscopy and endoscopic ultrasonography.^7^, ^8^, ^9^, ^10^, ^11^ We focused on factors such as an expansive appearance of the tumor, the presence of ulceration or erosion, converging folds toward the tumor, and poor extension of the tumor and the surrounding colorectal wall as endoscopic findings suggesting deep invasion of the submucosa by a colorectal LST. In lesions where pit patterns of the tumor site were evaluated on magnifying chromoendoscopy, we focused on the presence of an invasive pattern.^10^

**Figure 1:**
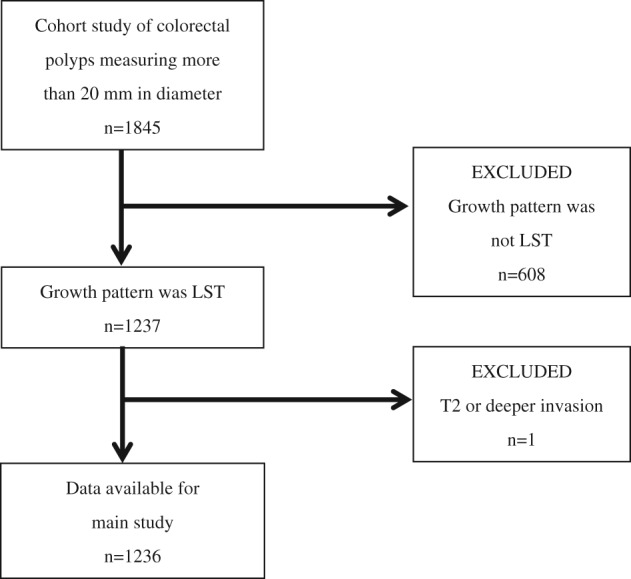
Flow diagram of colorectal laterally spreading tumor (LST) recruitment for the main study.

Among large colorectal tumors that were registered in a cohort study of colorectal polyps measuring more than 20 mm in diameter, 1236 lesions classified to have LST growth patterns were studied (Fig. 1). The inclusion criteria of the main study required LSTs measuring at least 20 mm in diameter that were histopathologically diagnosed to be adenomas or early cancers (Tis cancer or T1 cancer). LSTs that measured 20 mm or more in diameter with T2 or deeper histopathological invasion were excluded (Fig. 1).

#### *Validation study*

Among the institutions participating in the cohort study of colorectal polyps measuring more than 20 mm in diameter, a validation study of an independent series of colorectal LSTs that measured more than 20 mm in diameter and were resected endoscopically was conducted in two hospitals (K hospital and H hospital). The inclusion and exclusion criteria of the validation study were the same as those of the main study. As for the registration period and the number of registered lesions according to hospital, 131 consecutive lesions were registered in K hospital from January 2011 to December 2014, and 259 consecutive lesions were registered in H hospital from January 2003 to December 2009.

### *Survey variables*

For all colorectal LSTs measuring 20 mm or more in diameter that were registered in the main study and the validation study, we studied the patients' ages and genders, treatment procedures, lesion sites, maximum tumor diameters, LST subclassifications, and histological types (invasion depth in cases of cancer). Endoscopists in each hospital subclassified the LSTs based on colonoscopic findings, including the characteristics after spraying the lesions with indigo carmine dye. LSTs were subclassified according to the classification proposed by Kudo *et al*.^2^ LST‐G lesions were subclassified into LST‐G‐H and LST‐G‐M, and LST‐NG lesions were subclassified into LST‐NG‐F and LST‐NG‐PD.

### *Histopathological evaluations*

All resected samples were fixed in 10% formalin solution and sliced into specimens. Pathologists in each institution evaluated the size and the histological type of the lesions, as well as the depth of invasion for early cancers. The histological type of the adenomas or pathological Tis cancers and the invasion depth of the early cancers were classified according to the Japanese Classification of Colorectal Carcinoma.^12^ Cancer that was histopathologically confined to the mucosa was diagnosed as pathological Tis cancer. Cancer invading the submucosa was diagnosed as pathological T1 cancer.

### *Study end‐points*

In the main study, registered colorectal LSTs measuring 20 mm or more in diameter were subclassified to compare lesion sites, tumor diameters, and histological types among the subclassifications. We estimated whether the survey variables (predictive factors) could be used to predict the risks of pathological T1 cancer in patients with colorectal LSTs. Age, gender, LST subclassification, lesion site, and tumor diameter were used as predictive factors for evaluating the incidences of pathological T1 cancer associated with LSTs. Whether the predictive factors could be used to predict the risk of pathological T1 cancer associated with LSTs was analyzed. A validation study was performed to confirm the validity of the results obtained in the main study.

This main study and validation study were performed in accordance with the Declaration of Helsinki. The main study was approved by the ethics review committees in the JSCCR and each institution. The validation study was approved by the ethics review committees in the two participating hospitals. In the main study, all eligible patients were provided an explanation of the studies by their attending physicians and gave written informed consent. Because the validation study was an observational study of retrospectively collected data, it was difficult to explain the study to all patients and obtain written informed consent. Information about the study was therefore officially disclosed in a poster form at the participating hospitals after the study had been approved by the ethics review committee in each hospital. Informed consent was then obtained on an opt‐out basis from patients to ensure that they had an opportunity to refuse to participate in the study. The main study was registered in the University Hospital Medical Information Network, a public database in Japan, because the data were collected prospectively (UMIN000001642).

### *Statistical analysis*

Chi‐square tests and Fisher's exact probability tests were used to compare frequencies between the groups. *P* values of less than 0.05 were considered to indicate statistical significance. Continuous variables were expressed as means with standard deviations. In the main study, logistic regression analysis was performed to predict the risk of pathological T1 cancer associated with LSTs. Tumor size, growth pattern, and location and patient gender and age were included in the logistic model. The predictive value of each factor was calculated. The area under the curve (AUC) of the statistical model was calculated based on the area under the receiver operating characteristic curve. The external validity of the statistical model described in the main study was calculated using data obtained from the two hospitals in the validation study. Statistical analysis was performed using STATA14 software (StataCorp, Houston, TX, USA).

## Results

### *Patients and lesion characteristics*

The age and gender of the patients and the initial treatment regimens of the lesions are shown in Table 1. The mean age of the patients was 68 years, and males were more common than females in both the main study and the validation study. As for the initial treatment regimens, the number of lesions treated with ESD was higher than the number treated with EMR in the main study but was lower than the number treated with EMR in the validation study.

**Table 1:** Demographic characteristics of study subjects

	Main study	Validation study
Number of lesions	1236	356
Age (years), mean (SD)	68 (11)	68 (11)
Gender, *n* (%)		
Male	719 (58)	188 (53)
Female	517 (42)	168 (47)
Initial treatment, *n* (%)		
ESD	726 (59)	155 (44)
EMR	510 (41)	201 (56)

EMR, endoscopic mucosal resection; ESD, endoscopic submucosal dissection.

Histopathological characteristics are shown in Table 2. In both the main study and the validation study, LSTs were most commonly located in the rectum and the ascending colon and were least common in the descending colon. The mean tumor diameter was 35 mm in the main study and 34 mm in the validation study. The most common LST subtype was LST‐G‐M in both the main study and the validation study, and LST‐NG‐PD was the least common subtype. As for histological type, carcinomas accounted for about half of the lesions in both the main study and the validation study. Of the carcinomas, pathological T1 carcinomas accounted for 12% of the lesions in the main study and 9% of the lesions in the validation study.

**Table 2:** Histopathological characteristics of study subjects

	Main study (*n* = 1236), *n* (%)	Validation study (*n* = 356), *n* (%)
1. Location		
Rectum	319 (26)	92 (26)
Sigmoid	169 (14)	57 (16)
Descending	42 (3)	24 (7)
Transverse	241 (20)	54 (15)
Ascending	283 (23)	101 (28)
Cecum	182 (15)	28 (8)
2. Size		
20–29 mm	515 (42)	162 (46)
30–39 mm	322 (26)	97 (27)
≥40 mm	399 (32)	97 (27)
Mean (SD)	35 (17)	34 (16)
3. Growth pattern		
LST‐G‐H	314 (26)	84 (23)
LST‐G‐M	487 (39)	164 (46)
LST‐NG‐F	301 (24)	95 (27)
LST‐NG‐PD	134 (11)	13 (4)
4. Histology		
Adenoma	516 (42)	183 (51)
Tis carcinoma	567 (46)	141 (40)
T1 carcinoma	153 (12)	32 (9)

LST, laterally spreading tumor; LST‐G‐H, LST granular homogenous type; LST‐G‐M, LST granular nodular mixed type; LST‐NG‐F, LST nongranular flat‐elevated type; LST‐NG‐PD, LST nongranular pseudodepressed type.

### *LST subclassification and histopathological findings*

The locations of the LSTs in the main study were found to differ significantly according to the LST subclassification (*P* < 0.001). LST‐G‐H lesions were most commonly found in the ascending colon and the cecum, LST‐G‐M lesions in the rectum, and LST‐NG‐F lesions and LST‐NG‐PD lesions in the transverse colon (Table 3).

**Table 3:** Growth patterns of LSTs and histopathological findings in the main study

	LST‐G‐H (*n* = 314), *n* (%)	LST‐G‐M (*n* = 487), *n* (%)	LST‐NG‐F (*n* = 301), *n* (%)	LST‐NG‐PD (*n* = 134), *n* (%)
1. Location				
Rectum	54 (17)	219 (45)	28 (9)	18 (13)
Sigmoid	16 (5)	70 (14)	63 (21)	20 (15)
Descending	6 (2)	4 (1)	23 (8)	9 (7)
Transverse	34 (11)	37 (8)	108 (36)	62 (46)
Ascending	104 (33)	98 (20)	61 (20)	20 (15)
Cecum	100 (32)	59 (12)	18 (6)	5 (4)
2. Size				
20–29 mm	141 (45)	102 (21)	190 (63)	82 (61)
30–39 mm	91 (29)	114 (23)	77 (26)	40 (30)
≥40 mm	82 (26)	271 (56)	34 (11)	12 (9)
Mean (SD)	33 (14)	43 (2)	28 (9)	28 (9)
3. Histology				
Adenoma	190 (60)	159 (33)	133 (44)	34 (25)
Tis carcinoma	115 (37)	275 (56)	125 (42)	52 (39)
T1 carcinoma	9 (3)	53 (11)	43 (14)	48 (36)

LST, laterally spreading tumor; LST‐G‐H, LST granular homogenous type; LST‐G‐M, LST granular nodular mixed type; LST‐NG‐F, LST nongranular flat‐elevated type; LST‐NG‐PD, LST nongranular pseudodepressed type.

LSTs were subclassified into three groups according to the tumor diameter: lesions with a diameter of 20–29 mm, those with a diameter of 30–39 mm, and those with a diameter of 40 mm or greater. Tumor diameter differed significantly according to the LST subclassification (*P* < 0.001). The most common tumor diameter was 20–29 mm for LST subclassifications other than LST‐G‐M. An increase in tumor diameter was associated with a decreased number of lesions (Table 3). However, increased tumor diameters of LST‐G‐M lesions were associated with increasing numbers of lesions. More than half of all LST‐G‐M lesions had a tumor diameter of 40 mm or greater.

A significant difference was found when the histological types of LSTs were compared according to the LST subclassification (*P* < 0.001). LST‐G‐H lesions were associated with a high proportion of adenomas, and LST‐G‐M lesions were most commonly associated with pathological Tis carcinomas (Table 3). LST‐NG‐F lesions included nearly equivalent proportions of adenomas and pathological Tis carcinomas, and LST‐NG‐PD lesions included nearly equivalent proportions of pathological Tis carcinomas and pathological T1 carcinomas. The frequency of T1 cancers was the highest at 36% of LST‐NG‐PD lesions, followed by 14% of LST‐NG‐F lesions, 11% of LST‐G‐M lesions, and 3% of LST‐G‐H lesions.

### *Prediction of the risk of pathological T1 cancer*

Logistic regression analysis was performed to examine whether each predictive factor could predict the risk of pathological T1 cancer in the main study (Table 4). The risk of pathological T1 cancer was significantly related to tumor diameter and the subclassification of LSTs. The AUC indicating the predictive ability of the statistical model was high (0.743; 95% confidence interval, 0.702–0.784). The AUC indicating the external validity of the statistical model described above was 0.573.

**Table 4:** Risk prediction of pathological T1 cancer based on histopathological and epidemiological data

	Odds ratios	95% confidence intervals	
Size	1.02	1.01	1.03
Growth pattern (ref. LST‐G‐H)			
LST‐G‐M	3.13	1.48	6.66
LST‐NG‐F	8.36	3.84	18.24
LST‐NG‐PD	30.35	13.42	68.63
Location (ref. cecum)			
Ascending	1.13	0.56	2.30
Transverse	0.52	0.24	1.12
Descending	0.74	0.25	2.18
Sigmoid	0.77	0.35	1.67
Rectum	1.38	0.69	2.76
Gender (ref. female)			
Male	1.05	0.72	1.54
Age (unit: 1 year)	1.00	0.98	1.01

ROC curve: AUC = 0.743 (0.702–0.784).

External dataset: AUC = 0.573.

ROC, receiver operating characteristics; AUC, area under the curve; LST, laterally spreading tumor; LST‐G‐H, LST granular homogenous type; LST‐G‐M, LST granular nodular mixed type; LST‐NG‐F, LST nongranular flat elevated type; LST‐NG‐PD, LST nongranular pseudodepressed type.

## Discussion

Colorectal LST is not a macroscopic classification but is widely acknowledged because of its characteristic appearance and histopathological characteristics.^13^ The detection rate of colorectal LSTs on colonoscopy has been reported to be 1.3 and 4.5%.^14^, ^15^ LSTs have been reported to account for 5.8% of early colorectal cancers.^1^

Among colorectal LSTs, LST‐G lesions and LST‐NG lesions both predominantly develop in horizontal directions but have different histopathological characteristics. As for lesion site, LST‐G lesions most commonly arise in the rectum and the proximal colon. LST‐NG lesions are most commonly found in the transverse colon.^2^, ^16^ Compared with LST‐G lesions, LST‐NG lesions have been reported to be associated with a high incidence of cancer invading the submucosa despite a smaller tumor diameter.^17^, ^18^, ^19^ LST‐G and LST‐NG lesions are associated with different patterns of genetic abnormalities and are thus recognized as histopathologically distinct types of LSTs.^20^, ^21^, ^22^

In the main study, colorectal LSTs measuring 20 mm or greater in diameter were subclassified to compare histopathological characteristics among the subclassifications. Among the LSTs, LST‐G‐M lesions were most frequently found in the rectum, whereas other types of LSTs were most commonly located in the proximal colon. Many LST‐G‐M lesions were large, measuring 40 mm or greater in diameter. As for the histological type, the incidence of pathological T1 cancer was the highest among LST‐NG‐PD lesions. More than half of all LST‐G‐H lesions were adenomas, despite the fact that large lesions with a diameter of 20 mm or greater were studied. In the results of the main study, the incidence of pathological T1 carcinoma was only 3% of LST‐G‐H lesions. As for predictive factors for the risk of pathological T1 carcinoma, the results of logistic regression analysis showed that LST subclassification and tumor diameter were significantly related to the risk of pathological T1 carcinoma. The AUC of the statistical model was high. In particular, compared with LST‐G‐H lesions, an LST subclassification of LST‐NG‐PD was associated with an odds ratio of 30.35 for the risk of pathological T1 carcinoma, indicating high risk. In our study, a validation study was performed to evaluate the external validity of the statistical model used in the main study. A high AUC was obtained, confirming the validity of the results of the main study.

**Figure 2:**
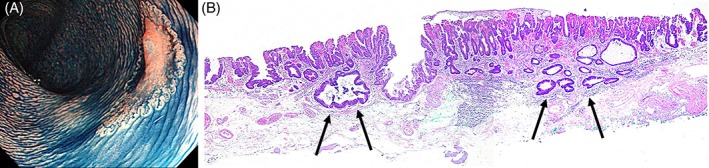
(a) Colonoscopic examination after spraying indigo carmine dye demonstrated a tumor with a poorly demarcated**,** basin‐like, shallow depression in the transverse colon. A laterally spreading tumor of nongranular pseudodepressed type was thus diagnosed. A protrusion was seen in the depression. (b) Histopathological findings of surgically resected specimens obtained by endoscopic submucosal dissection showing cancer with multifocal submucosal invasion (arrows).

An accurate preoperative evaluation of the invasion depth of early colorectal cancer plays an important role in determining the indication for endoscopic resection and in selecting the treatment procedure of choice. Among early colorectal carcinomas, endoscopic resection is indicated for the treatment of pathological Tis cancer because there is no risk of metastasis. The clinical management is the same as that for adenomas. Therefore, the distinction of pathological Tis cancer from adenoma based on endoscopic findings is considered to be not so meaningful. Among early colorectal cancers, pathological T1 cancer can metastasize to lymph nodes and other organs. Surgery should thus be considered. Therefore, before treatment, it is extremely important to accurately differentiate adenoma and pathological Tis cancer, indications for endoscopic treatment, from pathological T1 cancer,for which surgery should be considered. The results of the main study showed that not only tumor diameter but also LST subclassifications are important indicators that can be used to predict the invasion depth of LSTs. Investigators in other studies reported that the incidence of invasive carcinoma is the highest among LSTs with a subclassification of LST‐NG‐PD.^2^, ^23^, ^24^, ^25^, ^26^ Kudo *et al*.^2^ and Oka *et al*.^23^ reported that all large LST‐NG‐PD lesions with a diameter of more than 30 mm were pathological T1 carcinomas. In patients with LST‐NG‐PD lesions, cancer might multifocally invade the submucosa (Fig. 2a,b). When endoscopic resection is selected for the treatment of LST‐NG‐PD lesions, en bloc resection using procedures that can completely resect even large lesions, such as ESD, should be performed.^18^, ^23^, ^26^

More than half of the LST‐G‐H lesions were adenomas, and even if cancer was present, most cases were pathological Tis carcinomas. Other studies have reported that invasive carcinoma is rarely found among LST‐G‐H lesions.^2^, ^23^, ^25^, ^26^, ^27^, ^28^ Investigators in other studies have reported that the invasion depth of LSTs is related to tumor diameter.^2^, ^13^, ^23^ However, LST‐G‐H lesions were associated with a low incidence of pathological T1 cancer, irrespective of tumor diameter.^2^, ^23^, ^25^ Therefore, among LSTs, LST‐G‐H lesions were considered candidate lesions for piecemeal EMR regardless of the tumor diameter. Shigita *et al*.^29^ classified LST‐G into three subtypes according to the size and uniformity of granules and nodules on the tumor surface to compare the histological types among the subtypes. Many LST‐G lesions with a uniform surface were adenomas. About 10% of lesions with nodules that were 5–10 mm in diameter or with coarse nodules measuring 10 mm or greater were pathological T1 carcinomas. In our study, although the lesion size differed, 11% of LST‐G‐M lesions were pathological T1 carcinomas, indicating similar results. Therefore, ESD should be selected to endoscopically resect large LST‐G‐M lesions that have coarse nodules because it enables en bloc resection.

Our study had several limitations. First, a limitation of the main study and the validation study was that large colorectal LSTs that were evaluated to be free of submucosal deep invasion by endoscopists were resected endoscopically. The large colorectal LSTs that were examined in each study did not include lesions resected by surgical operation. Therefore, selection bias is considered a limitation of our study. The lesions that were suspected to have submucosal or deeper invasion and were resected surgically might have included lesions that were adenomas or pTis cancers on histopathological evaluation. A second limitation was that endoscopic resection is considered an acceptable procedure for the treatment of pathological T1 cancer confined to the shallow layer of the submucosa because such lesions are rarely associated with lymph node metastasis.^30^ However, in our study, the invasion depth of pathological T1 carcinomas, including the degree of submucosal invasion, could not be analyzed in detail. Although our study methods had these limitations, more than 1600 colorectal LSTs measuring 20 mm or more in diameter were included in the main and validation studies combined, and we believe that this is the first multicenter study to perform logistic regression analysis to determine predictors of the risk of pathological T1 carcinoma associated with large colorectal LSTs.

In conclusion, the results of logistic regression analysis indicate that the risk of pathological T1 carcinomas among large colorectal LSTs resected endoscopically can be predicted based on the LST subclassification and tumor diameter. The results of our study will hopefully facilitate the selection of the optimal treatment procedure for large colorectal LSTs.

## References

[1] Kudo S . Endoscopic mucosal resection of flat and depressed types of early colorectal cancer. Endoscopy. 1993; 25: 455–61. 8261988 10.1055/s-2007-1010367

[2] Kudo S , Takemura O , Ohtsuka K . Flat and depressed types of early colorectal cancers: from east to west. Gastrointest. Endosc. Clin. N. Am. 2008; 18: 581–93. 18674705 10.1016/j.giec.2008.05.013

[3] Oka S , Tanaka S , Saito Y *et al* Local recurrence after endoscopic resection for large colorectal neoplasia: a multicenter prospective study in Japan. Am. J. Gastroenterol. 2015; 110: 697–707. 25848926 10.1038/ajg.2015.96

[4] Takeuchi Y , Iishi H , Tanaka S *et al* Factors associated with technical difficulties and adverse events of colorectal endoscopic submucosal dissection: retrospective exploratory factor analysis of a multicenter prospective cohort. Int. J. Colorectal Dis. 2014; 29: 1275–84. 24986141 10.1007/s00384-014-1947-2

[5] Nakajima T , Saito Y , Tanaka S *et al* Current status of endoscopic resection strategy for large, early colorectal neoplasia in Japan. Surg. Endosc. 2013; 27: 3262–70. 23508817 10.1007/s00464-013-2903-x

[6] Wada Y , Kudo S , Tanaka S *et al* Predictive factors for complications in endoscopic resection of large colorectal lesions: a multicenter prospective study. Surg. Endosc. 2015; 29: 1216–22. 25159643 10.1007/s00464-014-3799-9

[7] Saitoh Y , Obara T , Watari J *et al* Invasion depth diagnosis of depressed type early colorectal cancers by combined use of videoendoscopy and chromoendoscopy. Gastrointest. Endosc. 1998; 48: 362–70. 9786107 10.1016/s0016-5107(98)70004-5

[8] Ikehara H , Saito Y , Matsuda T , Uraoka T , Murakami Y . Diagnosis of depth of invasion for early colorectal cancer using magnifying colonoscopy. J. Gastroenterol. Hepatol. 2010; 25: 905–12. 20546444 10.1111/j.1440-1746.2010.06275.x

[9] Kudo S , Rubio CA , Teixeira CR , Kashida H , Kogure E . Pit pattern in colorectal neoplasia: endoscopic magnifying view. Endoscopy. 2001; 33: 367–73. 11315901 10.1055/s-2004-826104

[10] Matsuda T , Fujii T , Saito Y *et al* Efficacy of the invasive/non‐invasive pattern by magnifying chromoendoscopy to estimate the depth of invasion of early colorectal neoplasms. Am. J. Gastroenterol. 2008; 103: 2700–6. 18853968 10.1111/j.1572-0241.2008.02190.x

[11] Kobayashi K , Kida M , Katsumata T *et al* Clinical role of endoscopic ultrasonography for the diagnosis of early colorectal cancer and selecting the treatment procedure. Dig. Endosc. 2003; 15: 298–305.

[12] Japanese Society for Cancer of the Colon Rectum . Japanese Classification of Colorectal Carcinoma, 8th edn. Tokyo: Kanehara & Co, 2013.

[13] Kudo S , Lambert R , Allen JI *et al* Nonpolypoid neoplastic lesions of the colorectal mucosa. Gastrointest. Endosc. 2008; 68 (Suppl. 4): S3–47. 18805238 10.1016/j.gie.2008.07.052

[14] Kaku E , Oda Y , Murakami Y *et al* Proportion of flat and depressed‐type and laterally spreading tumor among advanced colorectal neoplasia. Clin. Gastroenterol. Hepatol. 2011; 9: 503–8. 21440090 10.1016/j.cgh.2011.03.018

[15] Rotondano G , Bianco MA , Buffoli F , Gizzi G , Tessari F , Cipolletta L . The Cooperative Italian FLIN Study Group: prevalence and clinico‐pathological features of colorectal laterally spreading tumors. Endoscopy. 2011; 43: 856–61. 21826628 10.1055/s-0030-1256639

[16] Nishiyama H , Isomoto H , Yamaguchi N *et al* Endoscopic submucosal dissection for laterally spreading tumours of the colorectum in 200 consecutive cases. Surg. Endosc. 2010; 24: 2881–7. 20419319 10.1007/s00464-010-1071-5

[17] Toyonaga T , Man‐i M , Fujita T *et al* Retrospective study of technical aspects and complications of endoscopic submucosal dissection for laterally spreading tumors of the colorectum. Endoscopy. 2010; 42: 714–22. 20806155 10.1055/s-0030-1255654

[18] Uraoka T , Saito Y , Matsuda T *et al* Endoscopic indications for endoscopic mucosal resection of laterally spreading tumours in the colorectum. Gut. 2006; 55: 1592–7. 16682427 10.1136/gut.2005.087452 PMC1860093

[19] Tanaka S , Haruma K , Oka S *et al* Clinicopathologic features and endoscopic treatment of superficially spreading colorectal neoplasms larger than 20 mm. Gastrointest. Endosc. 2001; 54: 62–6. 11427843 10.1067/mge.2001.115729

[20] Noro A , Sugai T , Habano W , Nakamura S . Analysis of Ki‐ras and p53 gene mutations in laterally spreading tumors of the colorectum. Pathol. Int. 2003; 53: 828–36. 14629748 10.1046/j.1440-1827.2003.01564.x

[21] Hiraoka S , Kato J , Tatsukawa M *et al* Laterally spreading type of colorectal adenoma exhibits a unique methylation phenotype and K‐ras mutations. Gastroenterology. 2006; 131: 379–89. 16890591 10.1053/j.gastro.2006.04.027

[22] Sakai E , Fukuyo M , Matsusaka K *et al* TP53 mutation at early stage of colorectal cancer progression from two types of laterally spreading tumors. Cancer Sci. 2016; 107: 820–7. 26991699 10.1111/cas.12930 PMC4968595

[23] Oka S , Tanaka S , Kanao H , Oba S , Chayama K . Therapeutic strategy for colorectal laterally spreading tumor. Dig. Endosc. 2009; 21 (Suppl. 1): S43–6. 19691733 10.1111/j.1443-1661.2009.00869.x

[24] Horiuchi Y , Chino A , Matsuo Y *et al* Diagnosis of laterally spreading tumors (LST) in the rectum and selection of treatment: characteristics of each of the subclassifications of LST in the rectum. Dig. Endosc. 2013; 25: 608–14. 23369130 10.1111/den.12040

[25] Kim BC , Chang HJ , Han KS *et al* Clinicopathological differences of laterally spreading tumors of the colorectum according to gross appearance. Endoscopy. 2011; 43: 100–7. 21165823 10.1055/s-0030-1256027

[26] Fujishiro M , Yahagi N , Kakushima N *et al* Outcomes of endoscopic submucosal dissection for colorectal epithelial neoplasms in 200 consecutive cases. Clin. Gastroenterol. Hepatol. 2007; 5: 678–83. 17466600 10.1016/j.cgh.2007.01.006

[27] Imai K , Hotta K , Yamaguchi Y *et al* Should laterally spreading tumors granular type be resected en bloc in endoscopic resections? Surg. Endosc. 2014; 28: 2167–73. 24477937 10.1007/s00464-014-3449-2

[28] Saito Y , Fujii T , Kondo H *et al* Endoscopic treatment for laterally spreading tumors in the colon. Endoscopy. 2001; 33: 682–6. 11490384 10.1055/s-2001-16213

[29] Shigita K , Oka S , Tanaka S *et al* Clinical significance and validity of the subclassification for colorectal laterally spreading tumor granular type. J. Gastroenterol. Hepatol. 2016; 31: 973–9. 26601626 10.1111/jgh.13238

[30] Watanabe T , Muro K , Ajioka Y *et al* Japanese Society for Cancer of the Colon and Rectum (JSCCR) guidelines 2016 for the treatment of colorectal cancer. Int. J. Clin. Oncol. 2018; 23: 1–34. 28349281 10.1007/s10147-017-1101-6 PMC5809573

